# Malnutrition risk and frailty in head and neck cancer patients: coexistent but distinct conditions

**DOI:** 10.1007/s00405-022-07728-6

**Published:** 2022-12-09

**Authors:** Priya Dewansingh, Linda Bras, Lies ter Beek, Wim P. Krijnen, Jan L. N. Roodenburg, Cees P. van der Schans, Gyorgy B. Halmos, Harriët Jager-Wittenaar

**Affiliations:** 1 Research Group Healthy Ageing, Allied Health Care and Nursing, Hanze University of Applied Sciences, Petrus Driessenstraat 3, 9714 CA Groningen, The Netherlands; 2 Department of Otorhinolaryngology/Head and Neck Surgery, University Medical Center Groningen, University of Groningen, PO Box 30.001, 9700 RB Groningen, The Netherlands; 3 Department of Health Sciences, Faculty of Science, and Amsterdam Public Health Research Institute, Vrije Universiteit Amsterdam, Amsterdam, The Netherlands; 4 Faculty of Mathematics and Natural Sciences, University of Groningen, Groningen, The Netherlands; 5 Department of Oral and Maxillofacial Surgery, University Medical Center Groningen, University of Groningen, PO Box 30.001, 9700 RB Groningen, The Netherlands; 6 Faculty of Medical Sciences, University Medical Center Groningen, University of Groningen, PO Box 30.001, 9700 RB Groningen, The Netherlands; 7 Department of Rehabilitation Medicine, University Medical Center Groningen, University of Groningen, PO Box 30.001, 9700 RB Groningen, The Netherlands; 8 Department of Health Psychology Research, University Medical Center Groningen, University of Groningen, PO Box 30.001, 9700 RB Groningen, The Netherlands

**Keywords:** Malnutrition risk, Frailty, Head and neck cancer, PG-SGA, Oncology

## Abstract

**Purpose:**

Both malnutrition and frailty are associated with adverse treatment outcomes. Malnutrition (risk) and frailty are each commonly present in patients with head and neck cancer (HNC). However, their coexistence and association is unknown. Main goal of this study is to determine the coexistence of, and the association between malnutrition risk and frailty in patients with HNC.

**Methods:**

In this retrospective analysis on prospectively collected data, newly diagnosed patients with HNC, enrolled in the OncoLifeS databiobank were included. The Patient-Generated Subjective Global Assessment Short Form (PG-SGA SF) was used to assess malnutrition risk. The Groningen Frailty Indicator (GFI) was used to assess frailty status. Multivariate logistic regression analyses were performed, taking into account several patient- and tumor-related factors.

**Results:**

In total, 197 patients were included. Seventy-six patients (39%) had a medium or high malnutrition risk and 71 patients (36%) were frail. In 38 patients (19%), malnutrition risk coexisted with frailty. Patients with medium and high malnutrition risk were, respectively, 4.0 (95% CI 1.5–11.2) and 13.4 (95% CI 4.0–48.7) times more likely to be frail, compared to patients with low malnutrition risk. In turn, frail patients were 6.4 times (95% CI 2.6–14.9) more likely to have malnutrition risk compared to non-frail patients.

**Conclusions:**

Malnutrition risk and frailty frequently coexist but not fully overlap in newly diagnosed patients with HNC. Therefore, screening for both conditions is recommended.

**Supplementary Information:**

The online version contains supplementary material available at 10.1007/s00405-022-07728-6.

## Introduction

Malnutrition and frailty are serious health conditions, each commonly present in patients diagnosed with head and neck cancer (HNC) [1–3]. Both conditions are associated with adverse treatment outcomes, such as radiation-induced toxicity, postoperative complications, mortality, and poorer quality of life [4–8]. A combination of patient- and tumor-related factors typically associated with HNC is responsible for the high prevalence of malnutrition and frailty. For example, swallowing problems and pain in the upper aero-digestive tract as consequence of the tumor localization often lead to insufficient oral intake, unintentional weight loss and sarcopenia, especially in patients with mucosal HNC [1, 9–11]. Furthermore, tobacco use and alcohol use are the main risk factors for the development of HNC, but also cause comorbidities, like COPD and liver cirrhosis in these patients [12]. Each of these factors are associated with frailty.

In several patient populations, coexistence and interaction between malnutrition and frailty has been demonstrated, indicating that these conditions share common physical, social, and psychological risk factors [11, 13, 14]. Frailty has been defined as ‘a dynamic state affecting an individual who experiences losses in one or more domains of human functioning (physical, psychological, and social), which is caused by the influence of a range of variables and which increases the risk of adverse outcomes’ [15]. Frail individuals have an increased risk of losing independency in daily activities and an increased risk of mortality. Frailty can be potentially preventable and/or treatable, for example, by nutritional and physical activity interventions [16].

In The Netherlands, screening for malnutrition risk and frailty is routine in the hospital setting, to identify patients who may benefit from nutritional assessment and intervention before and during treatment [17]. To further improve the care pathways around the patients with HNC with multidimensional problems, we aimed to determine the coexistence of, and association between malnutrition risk and frailty.

## Materials and methods

### Study design and ethics

Since 2014, all newly diagnosed patients with HNC in the multidisciplinary head and neck oncological team of the University Medical Center Groningen (UMCG) were included in OncoLifeS, i.e., a data biobank which has been approved by the Medical Ethical Committee (UMCG METC approval 2010/109) and complies with the General Data Protection Regulation as stated by the European Union. Patients were enrolled after providing written informed consent. Data were selected from the OncolifeS data biobank, from patients included between August 2015 and January 2017. The study protocol was approved by the Scientific Board of OncoLifeS. The study is conducted in accordance with the current version of the Declaration of Helsinki.

### Study population and data collection

Two groups of patients were included. The first group consisted of patients with mucosal tumors in the upper digestive tract-like oral cavity, oropharyngeal, hypopharyngeal, and laryngeal malignancies. The second group consisted of patients with skin malignancies in the head and neck area. These two groups were distinguished, because the tumor often interferes with food intake in the group with mucosal tumors. During the diagnostic work-up, every patient filled out a set of questionnaires regarding demographic characteristics, comorbidities, smoking and alcohol use, socio-economic factors, cognitive functioning, frailty, and nutritional risk with the assistance of a healthcare professional.

### Variables

Malignancies were staged using the seventh edition of the TNM Classification of Malignant Tumors from the Union for International Cancer Control. Tumor type was categorized as follows: (1) mucosal, i.e., patients with mucosal squamous cell carcinoma of the oral cavity, oropharynx, and hypopharynx, larynx and (2) cutaneous, i.e., patients with a malignancy of the skin in the head and neck area. Comorbidities were assessed using the Adult Comorbidity Evaluation (ACE)-27, which categorizes patients with none, mild, moderate, or severe comorbidities based on 27 predefined items [18]. Cognitive functioning was assessed with the Mini-Mental State Examination (MMSE), in which cognitive impairment was defined by a score ≤ 24 [19].

### Malnutrition risk and frailty

The Dutch version of the Patient-Generated Subjective Global Assessment Short Form (PG-SGA SF) (version 3.7) was used to screen for malnutrition risk [20]. The PG-SGA SF is the patient component of the full PG-SGA, which is the reference method for nutritional assessment in patients with cancer [21]. The PG-SGA SF includes four boxes addressing weight history (Box 1), food intake (Box 2), nutrition impact symptoms (NIS), i.e., symptoms interfering with oral intake (Box 3), and activities and function (Box 4). PG-SGA SF total score ranges from 0 to 36. Patients with a score ≤ 3 were defined as low, ≥ 4 and ≤ 8 as medium, and ≥ 9 as high malnutrition risk [21, 22]. Patients with medium and high malnutrition risk were pooled and classified as ‘malnutrition risk’ for statistical analyses.

Frailty was assessed by the Groningen frailty indicator (GFI). The GFI consists of 15 questions regarding the following domains of life: daily activities, health problems, and psychosocial functioning, generating a score ranging from 0 to 15. Frailty was defined as a GFI score ≥ 4 [23].

### Statistical analysis

Continuous variables are presented as mean ± standard deviation (SD) for normally distributed variables, and as median with interquartile range (IQR) for skewed or ordinal variables. Normality was tested by the Kolmogorov–Smirnov test. The exact binomial Clopper-–Pearson estimation method was used for prevalence numbers and their 95% confidence interval (95% CI) of frail patients to have malnutrition risk, and patients with malnutrition risk to be frail. Multivariate data imputation was performed for missing data on variables to detect any meaningful differences with the results obtained after casewise deletion [24]. Univariate and multivariate binary logistic regression analyses were used to determine associations between malnutrition risk and frailty. Binary logistic regression analysis was performed separately using malnutrition risk or frailty as dependent outcome variable, respectively. For multivariate logistic regression analyses, the minimum Akaike Information Criterion (min AIC) was used to select and compare models that best predict new outcomes to determine the regression method that would most appropriately model the association between the outcome and the explanatory variables [25, 26]. Two tailed *p*-values were used with significance set at *p* < 0.05. Associations were presented as odds ratios (ORs) with 95% CIs. Statistical analyses were performed using IBM SPSS version 23.0 (SPSS Inc., Chicago, IL, USA). The Venn diagram, min AIC, and the multivariate logistic regression were produced using R Studio version 1.2.5019.

## Results

In total, 197 patients were included. Table 1 shows baseline characteristics of the patients. The mean age was 70.5 ± 11.5 years. The majority (68%) of patients was male. In total, 54 (27%) patients had medium malnutrition risk, 22 (11%) had high malnutrition risk, and 71 (36%) were frail.

**Table 1:** HNC study sample characteristics across malnutrition risk categories by PG-SGA SF

	*N*	Total group *N* = 197	Risk categories by PG-SGA SF		
			Low risk 0–3 points 121 (61)	Medium risk 4–8 points 54 (27)	High risk ≥ 9 points 22 (11)
Age, mean ± SD	197	70.5 ± 11.5	71.3 ± 11.5	71.7 ± 11.3	63.1 ± 9.7
Gender					
Male	197	134	82 (61)	39 (29)	13 (10)
Female		63	39 (62)	15 (24)	9 (14)
BMI, mean ± SD	194	25.9 ± 4.7	26.0 ± 4.1	26.5 ± 5.4	23.6 ± 5.5
Tumor type	197				
Mucosal		120	60 (50)	40 (33)	20 (17)
Cutaneous		77	61 (79)	14 (18)	2 (3)
Tumor localization	197				
Oral cavity		25	12 (48)	9 (36)	4 (16)
Oropharynx		41	12 (29)	18 (44)	11 (27)
Hypopharynx^a^		8	3 (38)	4 (50)	1 (13)
Supraglottic larynx		15	9 (60)	4 (27)	2 (13)
(Sub)glottic larynx		31	24 (77)	5 (16)	2 (7)
Skin		77	61 (79)	14 (18)	2 (3)
Classification for mucosal HNC	118				
Tis/T1/T2^a^		64	40 (63)	19 (30)	5 (8)
T3/T4/Tx		54	19 (35)	21 (39)	14 (26)
Classification for cutaneous HNC	74				
Tis/T1/T2^a^		54	42 (78)	10 (19)	2 (4)
T3/T4/Tx		20	16 (80)	4 (20)	0 (0)
Smoking	197				
Currently smoking		61	26 (43)	21 (34)	14 (23)
Never smoked/smoked in past		136	95 (70)	33 (24)	8 (6)
Alcohol, units/day, median (IQR)	174	1 (0–3)	1 (0–2)	1 (0–4)	2 (0–4)
Education	196				
Lower		92	53 (58)	29 (32)	10 (11)
Middle^a^		53	31 (58)	16 (30)	6 (11)
Higher		45	32 (71)	8 (18)	5 (11)
Other/unknown^a^		6	4 (67)	1 (17)	1 (17)
Marital status	197				
Single/widowed/divorced		66	40 (61)	19 (29)	7 (11)
Married/living together/not single^a^		131	81 (62)	35 (27)	15 (11)
Comorbidity^b^	181				
None/mild		96	65 (68)	21 (22)	10 (10)
Moderate/severe		85	42 (49)	31 (36)	12 (14)
Cognition^c^, median score (IQR)	197	28 (25–29)	28 (26–29)	27 (25–30)	29 (25–30)
Normal cognition		158	98 (62)	42 (27)	18 (11)
Impaired cognition		39	23 (59)	12 (31)	4 (10)
Frailty^d^	197				
Frail		71	33 (46)	25 (35)	13 (18)
Non-frail		126	88 (70)	29 (23)	9 (7)

Numbers are shown as *n* (%) unless reported otherwise

*SD* standard deviation, *IQR* interquartile range, *PG-SGA SF* Patient-Generated Subjective Global Assessment Short Form, *BMI* body mass index

^a^Percentages does not sum to 100, due to rounding

^b^Adult Comorbidity Evaluation 27

^c^Mini-Mental State Examination

^d^Groningen Frailty Indicator

### Coexistence of malnutrition risk and frailty

**Fig. 1:**
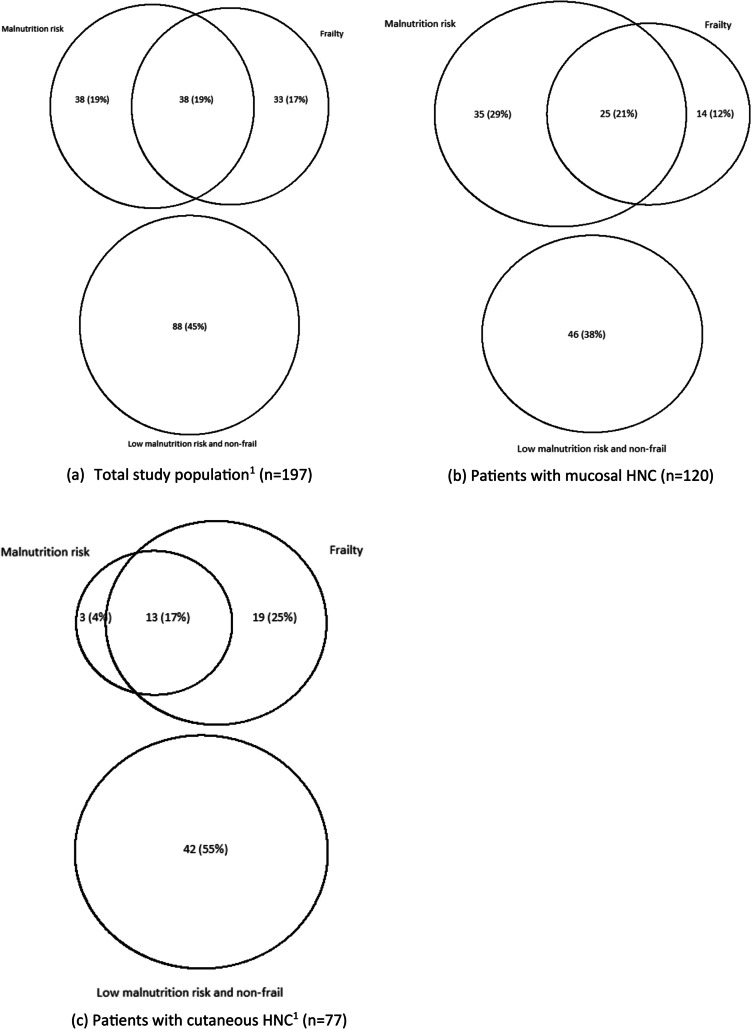
**a**–**c** Proportional Venn diagram of the coexistence of malnutrition risk and frailty in patients with head and neck cancer. *N* (%), ^1^Percentages do not sum to 100, due to rounding

Figure 1a–c shows proportional Venn diagrams of the coexistence of malnutrition risk and frailty. In total, 109 (55%) patients had malnutrition risk and/or were frail. Coexistence was present in 38 (19%) patients, while 38 (19%) patients had only malnutrition risk, and 33 (17%) patients were only frail. Almost half of the patients (*n* = 88, 45%) neither had malnutrition risk nor were frail.

Coexistence of malnutrition risk and frailty was found in 25 (21%) patients with mucosal HNC, and in 13 (17%) patients with cutaneous HNC. Solely malnutrition risk was present in almost one-third of patients with mucosal HNC (*n* = 35, 29%), and only in 3 (4%) patients with cutaneous HNC. Solely frailty was more often present in patients with cutaneous HNC (*n* = 19, 25%) compared to patients with mucosal HNC (*n* = 14, 12%). Moreover, absence of both malnutrition risk and frailty was more often present in patients with cutaneous HNC (*n* = 42, 55%) compared to patients with mucosal HNC (*n* = 46, 38%).

The exact binomial test with exact Clopper–Pearson 95% CI showed a prevalence of having malnutrition risk of 39% (95% CI 32–46%, *p* = 0.002) in frail patients. The prevalence of being frail was 36% (95% CI 29–43%, *p* < 0.001) in patients with malnutrition risk.

### Univariate and multivariate analyses of factors associated with malnutrition risk and/or frailty

Results of univariate analyses are presented in Tables 2 and 3. Age was significantly associated with frailty, but not with malnutrition risk. Patients with moderate to severe comorbidities significantly more frequently had malnutrition risk and frailty. Having a partner decreased the odds of being frail. Alcohol use was associated with higher odds of malnutrition risk, but with decreased odds of being frail. Patients with mucosal HNC were—not significantly—less often frail compared to patients with cutaneous HNC. However, patients with mucosal HNC had 4.8 times more often medium or high malnutrition risk compared to patients with cutaneous HNC.

**Table 2:** Univariate and multivariate modeling analyses of variables associated with frailty in head and neck cancer patients, *n* = 159

Frailty	Univariate model OR [95% CI]	*p*-value	Multivariate model AIC OR [95% CI]	*p*-value
Age^a^	1.06 [1.03–1.09]	**0.0006**	1.07 [1.03–1.12]	**< 0.001**
Smoking, yes	1.13 [0.56–2.23]	0.7309		
Sex, female	1.73 [0.85–3.52]	0.1280		
Comorbidity				
Moderate/severe	2.26 [1.17–4.43]	**0.0161**		
Marital status				
Married, living together, not single	0.31 [0.15–0.62]	**0.0010**	0.14 [0.05–0.35]	** < 0.001**
Education level				
Middle	0.44 [0.18–1.03]	0.0644		
High	2.07 [0.32–16.47]	0.4416		
Unknown	0.73 [0.45–1.16]	0.1873		
Alcohol^a^	0.78 [0.64–0.93]	**0.0108**	0.72 [0.58–0.87]	**0.002**
Cognition				
C ognitive impairment	0.15 [0.06–0.38]	**0.0001**	0.18 [0.05–0.60]	**0.007**
Tumor type				
Mucosal	0.64 [0.32–1.26]	0.194		
Medium malnutrition risk	2.35 [1.10–5.04]	**0.0278**	3.99 [1.50–11.20]	**0.007**
High malnutrition risk	3.79 [1.44–10.34]	**0.0076**	13.44 [4.04–48.71]	** < 0.001**

Bold indicates significant variables

*AIC* Akaike Information Criterion, *OR* odds ratio, *CI* confidence interval

^a^Continuous variable

**Table 3:** Univariate and multivariate modeling analyses of variables associated with malnutrition in head and neck cancer patients, *n* = 172

Malnutrition	Univariate model OR [95% CI]	*p*-value	Multivariate model AIC OR [95% CI]	*p*-value
Age^a^	0.98 [0.95–1.00]	0.0931		
Smoking, yes	3.29 [1.70–6.48]	**0.0005**	2.08 [0.95–4.61]	**0.068**
Sex, female	0.82 [0.41–1.62]	0.577		
Comorbidity				
Moderate/severe	2.37 [1.25–4.58]	**0.0092**		
Marital status				
Married, living together, not single	0.93 [0.50–1.75]	0.845	2.10 [0.93–4.99]	**0.084**
Education level				
Middle	0.59 [0.26–1.30]	0.1960		
High	0.73 [0.10–4.00]	0.7304		
Unknown	0.68 [0.43–1.06]	0.0935		
Alcohol^a^	1.15 [1.01–1.33]	**0.0457**	1.21 [1.02–1.45]	**0.029**
Cognition				
Cognitive impairment	1.01 [0.46–2.23]	0.981		
Tumor type				
Mucosal	4.82 [2.34–10.63]	**< 0.001**	4.23 [1.79–10.77]	**0.002**
Frailty, yes	2.75 [1.41–5.44]	**0.0032**	6.04 [2.63–14.86]	** < 0.001**

Bold indicates significant variables

*AIC* Akaike Information Criterion, *OR* odds ratio, *CI* confidence interval

^a^Continuous variable

Table 2 demonstrates that after correction for age, alcohol use, marital status, and cognition, patients with medium and high malnutrition risk were 4.0 and 13.4 times more likely to be frail compared to patients with low malnutrition risk, respectively. Vice versa, Table 3 shows that frail patients were 6.0 times more likely at risk of malnutrition compared to non-frail patients, after correction for smoking status, alcohol use, marital status, and tumor type.

### PG-SGA SF outcomes

The Supplementary file 1 shows scores on the PG-SGA SF for the study population and per frailty status.

## Discussion

This study shows that malnutrition risk and frailty considerably coexist in patients with newly diagnosed HNC. The prevalence of malnutrition risk or frailty alone is comparable to the prevalence of coexistence of malnutrition risk and frailty, i.e., 19% and 17%, respectively. Malnutrition risk is strongly positively associated with being frail. Medium and high malnutrition risk is related to 4.0 and 13.4 times more chance of being frail, respectively. In turn, frail patients are 6.0 times more likely to have medium or high malnutrition risk.

This is the first study investigating the coexistence and association between malnutrition risk and frailty in patients with HNC. In populations of older adults, the coexistence varies between 8 and 33% [13, 27–29]. The coexistence of almost 20% in our study is within this range. In line with previous findings in other populations, prevalence of both conditions separately was also considerable in our population [13]. Moreover, our results are in line with previous findings showing that older adults with malnutrition risk have a higher risk of being frail, and vice versa [30, 31]. Unfortunately, comparable studies in HNC populations are not available. Furthermore, comparison of our results with previous research is hampered due to use of different instruments for assessment of malnutrition risk and frailty.

The prevalence of frailty in our HNC study population is comparable with previous findings. Reported prevalence of frailty in HNC patients largely varies, i.e., between 7 and 75% [32–35], possibly depending on the methods used to determine frailty. Lowest percentages were found in retrospective population-based studies on hospitals’ discharge data, while highest percentages were found in studies using prospective multidimensional frailty instruments. Previous results from a comparable cohort of HNC patients showed a frailty prevalence of 40% [36]. Unfortunately, comparison of our findings on prevalence of malnutrition risk with previous studies is hampered, as previous studies in patients with HNC assessed malnutrition by the full PG-SGA rather than malnutrition risk by the PG-SGA SF. In those studies, prevalence of malnutrition varied between 31 and 44% [10, 37–39]. The association between malnutrition risk and frailty in patients with mucosal HNC is less strong compared to the association between malnutrition risk and frailty in our total study population. Despite comparable frailty prevalence in the total study population and the population with mucosal HNC, the latter showed a higher prevalence of malnutrition risk. It is likely that the prominently present swallowing problems in patients with mucosal HNC due to the tumor localization [40] more often result in malnutrition risk, independent of frailty.

The current study shows that alcohol consumption is associated with greater risk of developing malnutrition risk. However, this association is not shown for frailty, in which alcohol consumption even seems protective for being frail. This protective association between alcohol consumption and frailty was also found in a systematic review and meta-analysis [41]. Although the underlying mechanisms for a lower risk of frailty among alcohol consumers compared to non/past drinkers is not clear, it is possible that individuals who consume alcohol also have a stronger social network and stronger social support, which can prevent social isolation and therefore frailty [42].

A limitation of this study is the relatively small sample size, both for the whole study population and for the two subgroups, i.e., patients with mucosal tumors and patients with cutaneous tumors. As result, it was not possible to perform multivariate analyses per patient group. We countered this limitation by including the tumor type in the multivariate analysis.

Based on the current study, several clinical implications and recommendations can be formulated. First, malnutrition risk and frailty both need to be proactively screened for in patients with HNC, since both conditions not only coexist, but also separately occur in these patients. Although patients with mucosal HNC show the highest prevalence of malnutrition risk and frailty, screening for malnutrition risk in patients with complex cutaneous HNC is also relevant, since still one out of five of these patients has medium-to-high malnutrition risk. Screening for both conditions will identify different types of health-related problems per individual and may guide starting different interventions, e.g., nutritional interventions for patients with malnutrition or psychosocial support for frail patients, to optimize the patient’s pretreatment condition. Patients who remain malnourished and/or frail during and after cancer treatment are at risk of body tissue catabolism and wound healing disorders. These adverse advents can lead to a non-optimal treatment, making the already burdensome oncology treatment even harder for patients and can also lead to decreased overall survival [6, 40, 43]. Furthermore, previous studies in the same patient cohort as in the current study showed that medium malnutrition risk and frailty in patients undergoing surgery were both associated with postoperative complications [8]. Pretreatment medium-to-high malnutrition risk and frailty and were also associated with a decline in post-treatment quality of life [7, 44]. These findings also highlight the importance to screen patients with HNC for both conditions. Second, we recommend to screen for malnutrition risk and frailty to create awareness amongst healthcare professionals and patients [45] for potentially treatable factors. Frailty is a dynamic concept and the process of frailty can possibly be reversed [16]. Previous research has shown that nutritional status is prone to further deterioration during HNC treatment [46]. However, more research is needed to gain insight in the development of frailty during the course from diagnosis to rehabilitation and on the effect of specific supportive treatment that might lead to possibly reversing the patients’ frailty status.

## Conclusion

This study demonstrates considerable coexistence and an association between malnutrition risk and frailty in newly diagnosed patients with HNC, but also shows that both conditions considerably occur separately in these patients. Our findings highlight the importance of screening for both conditions in these patients at diagnosis. To potentially reverse malnutrition risk and frailty, targeted interventions are required.

## Supplementary Information

Below is the link to the electronic supplementary material.

Supplementary file1 (DOCX 16 KB)

## Data Availability

The datasets generated during and/or analysed during the current study are available from the corresponding author on reasonable request.

## Abbreviations

HNC: Head and neck cancer
COPD: Chronic obstructive pulmonary disease
UMCG: University Medical Center Groningen
ACE-27: Adult comorbidity evaluation 27
MMSE: Mini-mental state examination
GFI: Groningen Frailty Indicator
PG-SGA SF: Patient-Generated Subjective Global Assessment Short Form
TNM: Tumor, node, metastasis
NIS: Nutritional impact symptoms
SD: Standard deviation
IQR: Interquartile range
OR: Odds ratio
Min AIC: Minimum Akaike Information Criterion
95% CI: 95% Confidence interval
BMI: Body mass index
