# Supplementary material for: Malnutrition risk and frailty in head and neck cancer patients: coexistent but distinct conditions
Source: Eur Arch Otorhinolaryngol. 2022 Dec 9;280(4):1893–902. doi: 10.1007/s00405-022-07728-6 (PMC9988738; doi:10.1007/s00405-022-07728-6)
Supplement: Supplementary file 1 — Supplementary file1 (DOCX 16 KB) [file 405_2022_7728_MOESM1_ESM.docx]

**Supplementary file 1. PG-SGA SF characteristics in frail and non-frail head and neck cancer patients**

|  | N | Total population | Non-frail | Frail |
| --- | --- | --- | --- | --- |
| Box 1 Weight  % weight loss in one month, mean±SD  % weight loss in six months, mean±SD | 208  206 | 1.3±3.5 2.8±5.2 | 0.94±2.2 2.1±4.0 | 1.9±5.0  4.2±6.8 |
| Box 1 Weight  Decreased weight in last two weeks, n (%) | 217 | 41 (19) | 25 (12) | 16 (7) |
| Box 1 Weight score, median (IQR) | 219 | 0 (0-1) | 0 (0-0) | 0 (0-1) |
| Box 2 Food intake score, median (IQR) | 221 | 0 (0-1) | 0 (0-1) | 1 (0-1) |
| Box 3 NIS^a^ score, median (IQR) | 216 | 0 (0-3) | 0 (0-2) | 2 (0-4) |
| No problems eating, n (%) | 209 | 144 (69) | 108 (79) | 36 (50) |
| No appetite, just did not feel like eating, n (%) | 211 | 21 (10) | 7 (5) | 14 (19) |
| Nausea, n (%) | 211 | 3 (1) | 0 (0) | 3 (4) |
| Constipation, n (%) | 211 | 3 (1) | 2 (1) | 1 (1) |
| Mouth sores, n (%) | 211 | 30 (14) | 21 (15) | 9 (12) |
| Things taste funny or have no taste, n (%) | 211 | 6 (3) | 4 (3) | 2 (3) |
| Problems swallowing, n (%) | 211 | 37 (18) | 24 (17) | 13 (18) |
| Pain, n (%) | 211 | 42 (20) | 25 (18) | 17 (23) |
| Vomiting, n (%) | 211 | 2 (1) | 2 (1) | 0 (0) |
| Diarrhea, n (%) | 211 | 5 (2) | 1 (1) | 4 (6) |
| Dry mouth, n (%) | 211 | 12 (5) | 8 (6) | 4 (6) |
| Smells bother me, n (%) | 211 | 1 (1) | 7 (5) | 1 (1) |
| Feel full quickly, n (%) | 211 | 10 (5) | 3 (2) | 7 (10) |
| Fatigue, n (%) | 211 | 14 (7) | 3 (2) | 11 (15) |
| Other, n (%) | 211 | 12 (6) | 4 (3) | 8 (11) |
| Box 4 Activities and function score, median (IQR) | 218 | 0 (0-1) | 0 (0-1) | 1 (0-1) |
| PG-SGA SF score, median (IQR) | 221 | 2 (0-5) | 1 (0-4) | 4 (1-7) |

PG-SGA SF = Patient-Generated Subjective Global Assessment Short Form, SD = standard deviation, IQR=interquartile range, NIS = Nutrition Impact Symptom(s). ^a^ Patients could indicate more than one NIS
